# Activation of mTORC1 in chondrocytes does not affect proliferation or differentiation, but causes the resting zone of the growth plate to become disordered

**DOI:** 10.1016/j.bonr.2018.02.006

**Published:** 2018-02-24

**Authors:** Phillip T. Newton, Meng Xie, Ekaterina V. Medvedeva, Lars Sävendahl, Andrei S. Chagin

**Affiliations:** a Department of Physiology and Pharmacology, Karolinska Institutet, 17177 Stockholm, Sweden; b Department of Women's and Children's Health, Karolinska Institutet, Pediatric Endocrinology Unit, Karolinska University Hospital, 17176 Stockholm, Sweden; c Institute for Regenerative Medicine, Sechenov First Moscow State Medical University, Moscow, Russian Federation

**Keywords:** Chondrocyte, Growth plate, mTORC1, Tsc1, Knockout mice, Col2-Cre, Cre

## Abstract

There are several pitfalls associated with research based on transgenic mice. Here, we describe our interpretation and analysis of mTORC1 activation in growth plate chondrocytes and compare these to a recent publication (Yan et al., Nature Communications 2016, 7:11151). Both laboratories employed TSC1-floxed mice crossed with collagen type 2-driven Cre (Col2-Cre), but drew substantially different conclusions. It was reported that activation of mechanistic target of rapamycin complex 1 (mTORC1) *via* Tsc1 ablation promotes the hypertrophy of growth plate chondrocytes, whereas we observe only disorganization in the resting zone, with no effect on chondrocyte hypertrophy or proliferation. Here, we present our data and discuss the differences in comparison to the earlier phenotypic characterization of *TSC1* ablation in cartilage. Importantly, we detect Col2-Cre activity in non-cartilaginous tissues (including the brain) and discuss it in relation to other studies reporting non-cartilaginous expression of collagen alpha(1) II. Altogether, we conclude that mouse phenotypes following genetic ablation using Col2-Cre should be interpreted with care. We also conclude that activation of mTORC1 by *TSC1* ablation in postnatal chondrocytes with inducible Col2-Cre (Col2-CreERt) leads to disorganization of the resting zone but causes no changes in chondrocyte proliferation or differentiation.

## Highlights

• Ablation of Tsc1 using Col2-Cre causes severe developmental abnormalities.
• Col2-Cre is not specific to chondrocytes during early development.
• Mice develop normally when Tsc1 is ablated in chondrocytes postnatally.

## 1 Introduction

mTOR is a serine/threonine kinase activated by complex formation and this complex (mTORC) 1 coordinates anabolic (protein and lipid synthesis) and catabolic activities (autophagy) (Laplante and Sabatini, 2012). Both the function and sequence of mTOR have been highly conserved during evolution. For example, the yeast homolog of mTOR, TOR, regulates cell growth in response to nutrient supply (Laplante and Sabatini, 2012). The drosophila homolog, dTOR, also controls cell growth, as well as regulating body size (Oldham, 2011). With respect to mammals, mTORC1 signaling controls cell size in isolated systems, including liver, and in both skeletal and cardiac muscle (Lee et al., 2007). Genetic ablation of *MTOR* or *RPTOR* in the whole limb results in these structures being extremely small (Chen and Long, 2014).

We previously reported that activation of mTORC1 signaling stimulates bone growth *in vitro* (Newton et al., 2015) and aimed to explore its role *in vivo* by using Col2-Cre mice to ablate the gene encoding a key inhibitor of mTORC1, tuberous sclerosis 1 (*TSC1*). Although these mice displayed severe growth retardation starting after 2 weeks of age, we were dismayed that they also developed chronic wasting and seizures associated with premature death, which might be attributed to leakage of Cre in non-cartilaginous tissues. At the same time, a recent study based on this same model came to the conclusion that mTORC1 coordinates chondrocyte growth, proliferation and differentiation, in part *via* regulation of PTHrP (Yan et al., 2016).

Here, we suggest an alternative explanation to the phenotype described by Yan et al. (2016) based on actions of Tsc1 in cells other than chondrocytes. Furthermore, our present considerations are of key relevance to all experimental approaches involving tissue-specific gene ablation.

## 2 Results

1. Ablation of Tsc1 using Col2-Cre causes severe developmental abnormalities

We start by making direct comparisons below between our work and the similar experiments performed by Dr. Yan et al. (2016), we refer to our figures in **bold letters** and those of Yan and colleagues in *italics*.

**Fig. 1:**
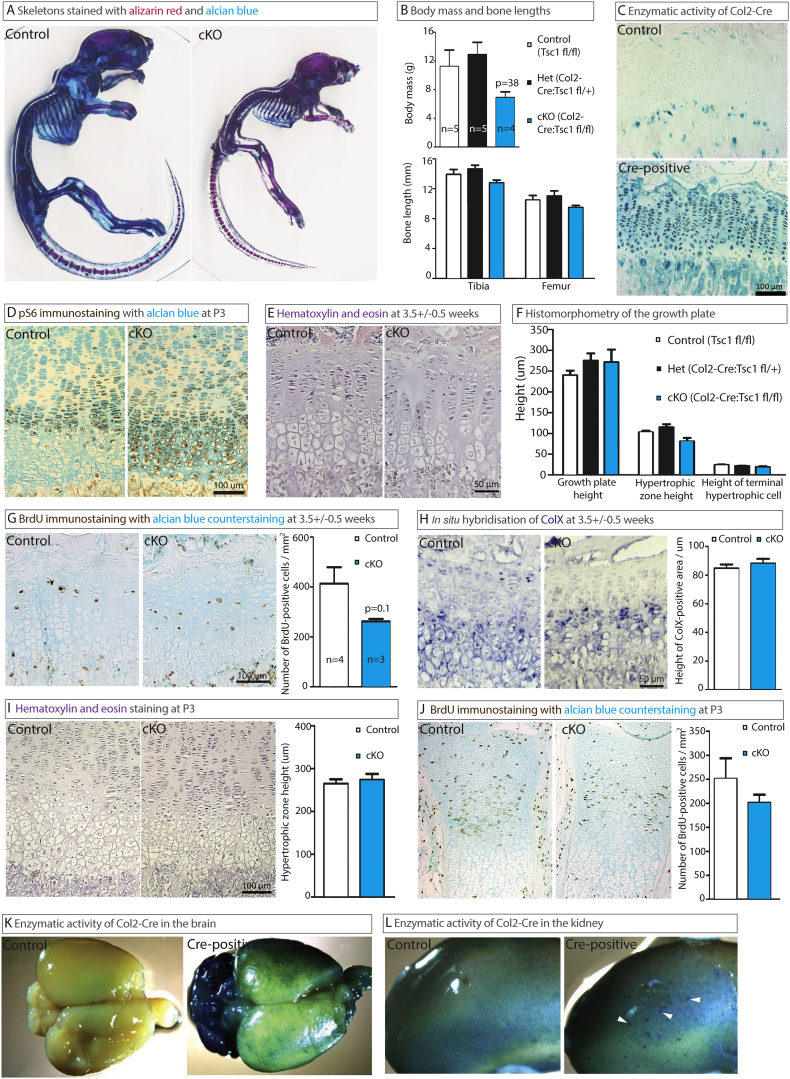
Col2-Cre:*Tsc1* fl/fl mice develop abnormally, but their bones are relatively normal. (**A**) Skeletal preparations from one-month-old mice stained with alcian blue and alizarin red. (**B**) The body mass and bone length of mice at 3.5 ± 0.5 weeks of age. (**C**) The enzymatic activity of Cre in sections of growth plate from the three-week-old progeny of Rosa26 reporter mice crossed with Col2-Cre:*Tsc1* fl/fl mice (upper panel – no Cre; lower panel - Cre-positive). (**D**) Immunohistochemical staining for phosphorylated S6 in the growth plate of 1-day-old mice. (**E**) Staining of sections of the growth plate of 3.5 ± 0.5-week-old mice with hematoxylin and eosin (H&E) for (**F**) morphometric analysis. (**G**) Immunohistochemical quantification of BrdU (injected 2 h prior to sacrifice) and quantification in 3.5 ± 0.5-week-old mice. (**H**) *In situ* hybridization and quantification of COL10A1 transcription in the growth plates of mice at 3.5 ± 0.5 weeks of age. (**I**) H&E staining for histomorphometry of the growth plates of three-day-old mice. (**J**) Immunohistochemical quantification of BrdU (injected into 2 ± 1-day old mice 2.25 ± 0.25 h prior to sacrifice). (**K**, **L**) The enzymatic activity of Cre in the whole brain and kidney of Col2-Cre:Rosa26 reporter mice, as determined by staining with X-gal at one month of age. In this figure, all controls are Col2-Cre-negative mice and all cKO mice have the genotype Col2-Cre:*Tsc1* fl/fl, unless otherwise stated. The values presented are means ± SEM. No significant differences were detected between control and cKO mice (for G–J) utilizing either one-way ANOVA or an unpaired *t*-test.

Like Yan et al. (2016), we ablated *TSC1* in murine chondrocytes utilizing non-inducible Collagen2-driven Cre (Kobayashi et al., 2002) and observed similar growth retardation in the resulting mice (Fig. 1**A** and **B** *versus Fig*. *3c* and *6b* (Yan et al., 2016)). Our ablation was confirmed by PCR (not shown), as well as in rosa26-LacZ reporter mice (Fig. 1**C**), which demonstrated 99.91% recombination efficiency in the growth plate (2362 cells from two mice). S6 activity in the growth plate of Tsc1cKO mice was elevated (Fig. 1**D**, similar to *Fig*. *3b* (Yan et al., 2016)). Clearly, our *TSC1* ablation was successful. Col2-Cre:Tsc1 fl/+ mice were used as a control for the effect of Col2-Cre recombinase itself (Fig. 1**B** and **F**). As it showed no phenotype (Fig. 1**B** and **F**), which is in line with previous observations by us and others (Kobayashi et al., 2002; Bouderlique et al., 2015; Vuppalapati et al., 2015), we did not perform further analysis of this specific genotype.

**Fig. 2:**
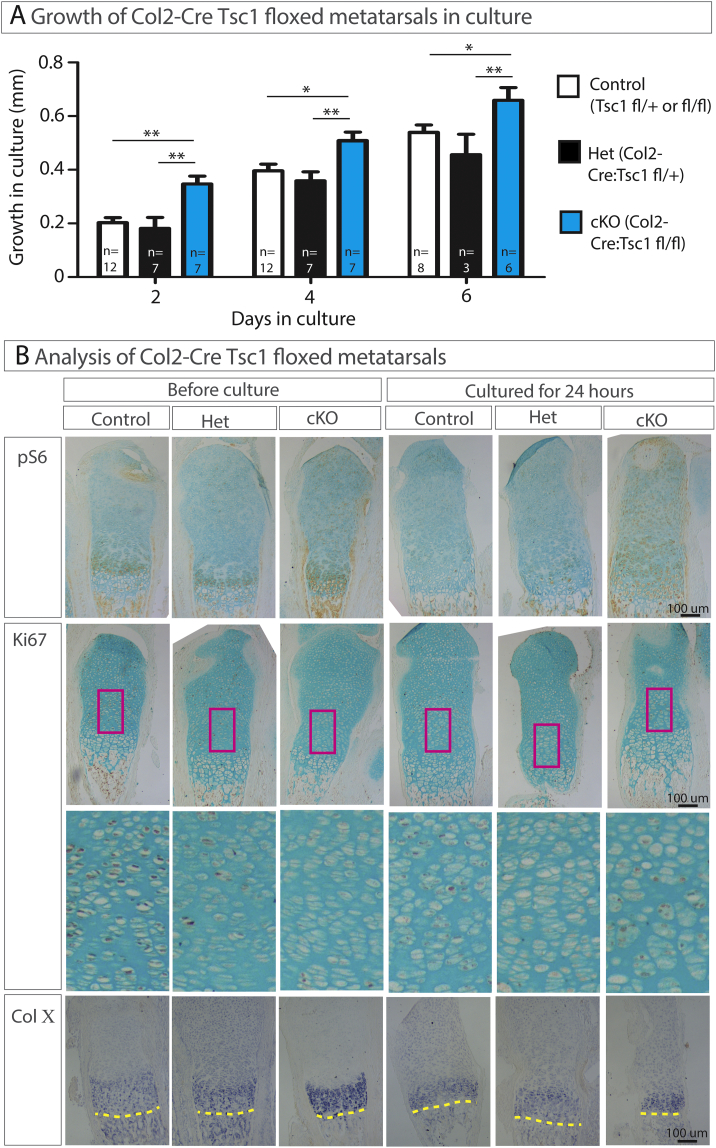
Col2-Cre:*Tsc1* fl/fl metatarsals have a growth advantage *ex vivo*. (**A**) Growth curves of metatarsals collected from three-day old mice and cultured for six days *ex vivo*. (**B**) Metatarsals were collected immediately before culture or after one day in culture (contralateral controls), then stained for pS6, Ki67 (immunohistochemistry) or COL10A1 (*in situ* hybridization). For Ki67 staining, the region directly above the hypertrophic zone is expanded (pink box). For COL10A1 staining, the yellow dashed line denotes the border between the cartilage and the primary spongiosa. (For interpretation of the references to color in this figure legend, the reader is referred to the web version of this article.)

**Fig. 3:**
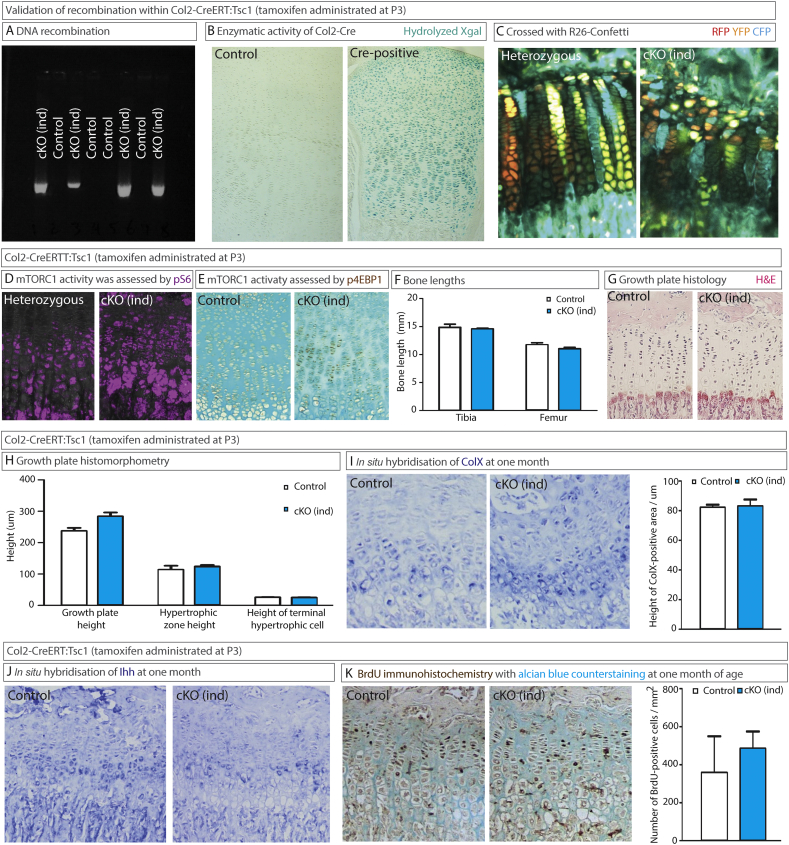
Postnatal ablation of Tsc1 in mice does not affect cell proliferation or hypertrophy in the growth plate, but leads to disorganization of the resting zone. Unless otherwise stated, in all these images, data on control (Col2-CreERT-negative) mice are shown to the left and on Tsc1cKO^ind^ (Col2-CreERT:*Tsc1* fl/fl) mice to the right. To achieve recombination, all animals were injected with tamoxifen on P3 and sacrificed at 8 days (A, B, E) or 1 month of age (C, D, F–K). (**A**) Cartilage was isolated from the elbows, DNA extracted, and PCR performed to identify the product of *Tsc1* recombination. The bands are seen exclusively in the Cre-positive *Tsc1* fl/fl animals. (**B**) The enzymatic activity of Cre in sections of growth plate from Col2-CreERT:Rosa26 reporter mice was assessed by staining with X-gal. (**C**) The R26-Confetti reporter strain (in which Cre recombination leads to the expression of different fluorescent proteins, in a stochastic manner) was crossed with Col2-CreERt and *Tsc1*-floxed mice and the progeny injected with tamoxifen on P3 and analyzed at 1 month of age. Col2-CreERt:*Tsc1* fl/+: R26-Confetti on the left and Col2-Cre:*Tsc1* fl/fl: R26-Confetti on the right panel. (**D**) The level of phosphorylated S6 in cKO^ind^ mice was higher than in the control mice (1 month of age). (**E**) The level of phosphorylated 4EBP1 in cKO^ind^ mice was higher than in the control mice (**F**) Lengths of the tibia (t) and femur (f) are shown at one month of age. (**G**, **H**) Sections of the growth plate were stained with H&E staining and morphometric analysis was conducted. (**I**) Transcription of COL10A1 and (**J**) Ihh expression in the growth plate, as detected by *in situ* hybridization. (**K**) Immunohistochemical quantification of BrdU (injected 2 h prior to sacrifice). All values presented are means ± SEM (n = 3–8). No significant differences were detected between control and cKO mice utilizing an unpaired *t*-test (F, H, I and K).

In contrast to Yan et al. (2016), we found no significant changes in the height of the growth plate or of the hypertrophic zone (Figs. 1**E**, **F** and 2**B** *versus Fig*. *3e* (Yan et al., 2016)), the level of BrdU incorporation (Fig. 1**G** *versus Fig*. *4b* (Yan et al., 2016)) or the expansion of collagen X-producing hypertrophic chondrocytes (Figs. 1**H**, 2**B** *versus Fig*. *5a* (Yan et al., 2016)). Yan and co-workers reported increased hypertrophy of chondrocytes in the rib upon Tsc1 ablation (*Fig*. *4a* (Yan et al., 2016)). We did not analyze the ribs, but the size of terminal hypertrophic chondrocytes in the proximal tibia was not changed upon Tsc1 ablation (Fig. 1**F**). However, we noticed some irregularity in the resting zone of the adult Tsc1cKO mice (Fig. 1**E**), similar to that shown by Yan and co-workers (*Fig*. *3d* (Yan et al., 2016)).

Our Tsc1cKO mice began dying at approximately 4 weeks of age (not shown), which was also the case in the Yan study (*Fig*. *6a* (Yan et al., 2016)). For ethical reasons, we had to sacrifice these mice at this point, since they began to suffer severely, developing seizures and chronic wastage.

In general, the growth retardation we observed was in contrast to our recent report that activation of mTORC1 signaling stimulated bone growth *in vitro* (Newton et al., 2015). However, we found that when cultured *ex vivo* metatarsal bones from Tsc1cKO mice grew longer than control bones (Fig. 2**A**), which could be detected after the first 24 h in culture (52% increase in growth: control bones grew 150 ± 13 μm; cKO bones grew 229 ± 19 μm, p = 0.0016, n = 18 and this growth increment was sustained over the culture period) albeit no changes in cell proliferation or the expression of collagen type X was observed (Fig. 2**B**). No difference in bone length was observed prior to culture (control = 2.711 ± 0.046 mm *versus* cKO = 2.700 ± 0.052 mm, p = 0.9237, n = 18). We concluded that additional factors are required for stimulation of mTOR-dependent growth *in vivo* and that the general growth phenotype displayed by Tsc1cKO mice may not be related to the role of Tsc1 in chondrocytes.

2. Col2-Cre activity in non-cartilaginous tissues

The suffering experienced by our Col2-Cre:TSC1 flox/flox mice was unexpected, since the growth plate appeared phenotypically normal at both 3 days (Fig. 1**I** and **J**, similarly to the P0 time-point in *Fig*. *3d* (Yan et al., 2016)), 16 days (Table 1) and 3.5 weeks of age (Fig. 1**A**–**H**). Usually, only severe malformations of cartilage, such as in the rib cage, skull or tooth, are lethal to mice and no such malformations were detected in our Tsc1cKO animals (not shown). On the other hand, it has been reported that during early development Collagen 2 is expressed in the liver, lingual epithelium of the tongue, salivary gland, adrenal cortex, apical ectodermal ridge (Lui et al., 1995), fetal brain, eye and heart (Cheah et al., 1991), as well as that Col2-Cre can also target other tissues, such as the head mesoderm and notochord (from as early as E8.75), cranial mesenchyme (from E11.5), submandibular glands (from E14.5) (Ovchinnikov et al., 2000), epithelial cells of the kidney, pancreas, lungs, intestine and ovaries (Kolpakova-Hart et al., 2008), osteoblasts and osteocytes (Vuppalapati et al., 2015), mesenchymal cells and the central nervous system (Long et al., 2001). Our observation of β-galactosidase reporter activity in the brain and kidney of Col2-Cre:Rosa26R:*Tsc1* fl/+ mice (Fig. 1**K** and **L**) constitutes further evidence for the ability of non-inducible Col2-Cre to target non-cartilaginous tissues. In this context it is interesting to emphasize that neuronal ablation of *Tsc1* leads to postnatal growth retardation, seizures and drastically reduced survival after 3 weeks of age (Meikle et al., 2007), all of which can be rescued by rapamycin (Meikle et al., 2008). Thus, neuronal ablation of Tsc1 closely mimics the phenotype observed upon ablation of Tsc1 with Col2-Cre.

3. Mice with postnatal chondrocyte-specific ablation of Tsc1 develop normally

**Table 1:** Effect of Tsc1 ablation on bone length and growth plate histology at 16 days of age.

	Cre negative Tsc1 fl/fl	Col2-Cre positive Tsc1 fl/fl	p-Value
	Value (n)	Value (n)	
Body mass (g)	6.00 ± 0.14 (5)	6.03 ± 0.07 (3)	0.867
Tibial length (mm)	11.88 ± 0.11 (5)	11.52 ± 0.08 (3)	0.070
Femoral length (mm)	8.45 ± 0.13 (5)	8.58 ± 0.02 (2)	–
Growth plate height (um)	305.2 ± 17.9 (3)	324.5 ± 30.8 (3)	0.616
Hypertrophic zone height (um)	133.0 ± 7.0 (3)	142.2 ± 10.8 (3)	0.511
Terminal hypertrophic cell size (um)	24.0 ± 0.9 (3)	24.8 ± 0.6 (3)	0.532

Since Col2-Cre was active not only in chondrocytes, we reasoned that the death of Tsc1cKO mice could be due to ablation of *TSC1* in other tissues and that the skeletal effects might be secondary. To test these proposals, we employed the inducible Col2-CreERt system (Nakamura et al., 2006) for postnatal ablation of *TSC1* in the growth plate, which avoids any activity of Cre in non-cartilaginous tissues during embryonic period. The Col2-CreERt:*Tsc1* flox/flox (Tsc1cKO^ind^) mice obtained were injected with tamoxifen on postnatal day 3 in accordance with protocols established previously (Chagin et al., 2014). As we have extensively characterized this Col2-CreERt strain before and never observed the effect of the CreERt transgene *per se* (Chagin et al., 2014; Hirai et al., 2011; Kaucka et al., 2016; Kaucka et al., 2017; Li et al., 2017), we presented analysis of CreERt positive controls in Fig. 3C and D only. Recombination of *TSC1* DNA was confirmed by PCR (Fig. 3**A**) and sequencing (not shown) and CreERt activity was confirmed in two reporter strains: Rosa26-LacZ (Fig. 3**B**) and R26-Confetti (Fig. 3**C**), the latter with growth plate chondrocytes exhibiting a recombination efficiency of 66.36% (3921 cells analyzed from 4 mice) at the dose of tamoxifen used throughout. Activation of mTORC1 signaling was confirmed by elevated phosphorylation of S6 by 4.1 folds (fluorescent intensity 0.0033 ± 0.0005 in heterozygotes *versus* 0.0135 ± 0.0033 arbitrary units, p < 0.05, n = 3; see also Fig. 3**D**) as well as phosphorylation of 4EBP1 (Fig. 3**E**), two well-known readouts of mTORC1 activity. No recombination occurred without tamoxifen injection as analyzed in Col2-CreERt:R26-Confetti mice (data not shown).

These mice developed normally (not shown), with normal bones (Fig. 3**F**) and no alterations in chondrocyte hypertrophy (Fig. 3**G** and **H**), the expression of collagen type X (Fig. 2**I**), Ihh (Fig. 2**J**) or cell proliferation (Fig. 3**K**). However, the height of the growth plate was significantly increased (p < 0.05, Fig. 3**F**, **G**), as the result of an expansion of the resting zone (34 ± 3 *vs* 58 ± 8 μm, p = 0.108; n = 4 (heterozygous) or 8 (homozygous mutant)). Indeed, irregularities in the resting zone chondrocytes could be seen in both non-inducible (Fig. 1**E**) and inducible (Fig. 3**G**) Tsc1cKO strains, particularly striking when *Tsc1* ablation was performed in the clonal R26-Confetti reporter strain (Fig. 3**C**).

On the basis of our experiments with two different Cre strains, we conclude that ablation of *TSC1* does not affect chondrocyte proliferation or differentiation, in contrast to the conclusion by Yan et al. (2016). We believe that side-effects associated with Col2-Cre leakage in combination with the general suffering/dying of Tsc1cKO mice after 4 weeks of age (*Fig*. *6a*), the time-point at which Yan and colleagues performed most of their phenotypic analyses (Figs. 3, *4*, *5*, *6*, *S3*, *S4*, *S6*) (Yan et al., 2016), may explain their findings.

## 3 Discussion

Based on our previous observations (Newton et al., 2015), we had hypothesized that elevation of mTORC1 activity in the growth plate would stimulate chondrocyte hypertrophy and thereby increase longitudinal growth. However, when we ablated Tsc1 in chondrocytes using Col2-Cre and carefully assessed the growth plates of these mice, we found no changes in chondrocyte differentiation or proliferation, in contrast to the report by Yan and colleagues (Yan et al., 2016).

One potential explanation for these discrepancies is that the mice employed in both these studies were of mixed genetic backgrounds (personal correspondence with Dr. Bai). Additionally, both studies used different Cre strains: we used one produced by Dr. Johnson (Kobayashi et al., 2002) whereas Dr. Bai's laboratory used the strain obtained as a gift from Dr. Yang (presumably described here (Yan et al., 2016; Hao et al., 2002)). Thus, the phenotype of the two strains (Col2-Cre^Johnson^:Tsc1 fl/fl and Col2-Cre^Yang^:Tsc1 fl/fl) can conceivably be different. However, since the gross phenotypes of the Col2-Cre^Johnson^:Tsc1 fl/fl and Col2-Cre^Yang^:Tsc1 fl/fl mice are the same it seems likely that the mechanisms by which Tsc1 causes growth retardation and death are also similar. Interestingly, ablation of Tsc1 in neurons (Tsc1cKO-neuro) pheno-copied Col2-Cre:Tsc1 fl/fl mice (Tsc1cKO-cartilage), with similar growth retardation, thoracic lordosis and increased mortality from 3 weeks of age (Meikle et al., 2008; Carson et al., 2012). Tsc1cKO-neuro has reported seizures, which are also observed in our Tsc1cKO-cartilage mice (not reported by Yan et al.). This observation, combined with the absence of such features as growth retardation, seizures and death upon postnatal Tsc1 ablation (Tsc1cKO^ind^) suggest that the growth plate phenotype can be secondary to non-specific activity of Col2-Cre line in the brain during development. Activity in other non-cartilaginous tissues can also cause the described phenotype, since the expression of alpha 1(II) collagen during embryonic development was demonstrated in the heart, epidermis, inner ear, notochord, sclera of developing eye, neural retina, the corneal and conjunctival epithelia, proliferative ventricular cells of the forebrain and midbrain and the cervical spinal cord (Cheah et al., 1991). The activity of Col2-Cre in non-cartilaginous tissue has also been well reported (Vuppalapati et al., 2015; Ovchinnikov et al., 2000; Kolpakova-Hart et al., 2008). Accordingly, the data should always be interpreted carefully and although the Cre-lox approach is one of the most powerful tools for examining the functions of gene products in target tissues, expression of Cre in non-target cells is a serious drawback and must always be taken into account.

It is clear that mTORC1 is important for limb development (Chen and Long, 2014; Wu et al., 2017; Phornphutkul et al., 2009). However, our present findings with Tsc1cKO^ind^ mice indicate that elevated mTORC1 activity in chondrocytes is not in itself sufficient to disrupt their functionality or proliferative activity. We did, however, observe that the resting zone in Tsc1cKO^ind^ mice was disorganized, which is also apparent in the non-inducible model. Accordingly, this disorganization, confirmed in experiments with both non- and inducible Col2-Cre strains, is probably due to direct activation of mTORC1 in chondrocytes. Altogether, these observations suggest that mTOR-signaling pathway is not essential, but rather modulates the growth plate chondrocytes. In this regard it is interesting to point out that ablation of mTOR in the adult cartilage poses no histological phenotype in the growth plate (Zhang et al., 2015).

In conclusion, and in contrast to a recent report, elevated mTORC1 signaling in chondrocytes does not alter the proliferation or differentiation of growth plate chondrocytes. The described phenotype is likely caused by aberrant Cre-activity in other non-cartilaginous tissues early in development.

## 4 Materials and methods

### 4.1 Mouse strains

All animal work was approved by the Ethical Committee on Animal Experiments (Stockholm North Committee/Norra Djurförsöksetiska Nämd) and conducted in accordance with The Swedish Animal Agency's Provisions and Guidelines for Animal Experimentation.

The Col2-Cre strain (Kobayashi et al., 2002) expresses Cre recombinase under the transcriptional control of the Collagen type 2a1 promoter (Col2a1) (Schipani et al., 2001). The Col2a1 gene is active in chondrocytes, which facilitates expression of high levels of Col2-Cre in chondrocytes (Schipani et al., 2001).

In the Col2-CreERt strain, developed by Susan Mackem (NIH), expression of CreERt protein is controlled by Col2a1(Nakamura et al., 2006); Cre-mediated DNA recombination is dependent on tamoxifen in both of these strains.

Rosa26-lacZ is a reporter mouse strain in which upon Cre-mediated recombination, beta galactosidase is expressed, allowing visualization of cells in which Cre has acted (Soriano, 1999) (JAX#003309).

In the Tsc1-floxed mice (JAX#005680) were developed by Kwiatkowski and colleagues, Cre-mediated recombination excises the essential exons 17 and 18 (Kwiatkowski et al., 2002).

Rosa26-Confetti (referred to as Confetti, and obtained from the laboratory of Hans Clevers (Hubrecht Institute) is a reporter mouse strain that contains the brainbow 2.1 construct (Livet et al., 2007). Upon Cre-mediated DNA recombination, one of four different fluorescent proteins (nuclear green, cytoplasmic red, cytoplasmic yellow and membrane-bound cyan) is expressed in a stochastic manner from each allele, allowing clonal identification (Snippert et al., 2010). Importantly, in our system we almost never (<1%) observed the appearance of green fluorescent of confetti colors.

The Col2-CreERt strain was crossed with Tsc1-floxed and Confetti mice to obtain inducible, chondrocyte-specific loss of Tsc1 in the developing epiphyseal cartilage (Tsc1cKO^ind^). Gene ablation/tracing was induced on P3 by IP injection of 500 μg tamoxifen into each mouse. All control mice were littermates of the cKO mice.

### 4.2 Confocal microscopy

Confocal imaging was performed with a Zeiss LSM710 fluorescence microscope. The images displayed are of maximal projection. Contrast and brightness have been altered to improve visualization; where test and control are included, adjustments were made in an identical manner.

### 4.3 Immunofluorescence

Immunofluorescence was conducted to detect pS6 using tissues fixed for 6 h in 4% PFA/PBS, without antigen retrieval. Briefly, after blocking with 3% horse serum in PBS-Triton (PBS + 0.1% Triton), slides were incubated with primary antibody (Cell Signaling Technology, 4858S) overnight at 4 °C, washed with PBST (PBS + 0.1% Tween 20) and subsequently incubated with secondary antibody (Alexa fluor 647-conjugated Donkey anti-Rabbit IgG; Jackson laboratories, 711 605 152) for 1 h at room temperature and thereafter protected from light. In this particular experiment, mice were injected with a dose of 0.4 mg of tamoxifen on P3 and tissues collected on P40.

### 4.4 Quantification of fluorescent signal

Z-stack images were collected by confocal microscopy using the same settings for each slide, and maximum projection images obtained. ImageJ software (NIH, Bethesda, U.S.A.) was used for the digital analysis.

Firstly, to outline the growth plate precisely Freehand Selection tool and the “Edit>Clear Outside” function was used. The area of the growth plate was determined using the “Analyze>Measure” function (“Area” must be selected in the “Analyze>Set Measurements…” function). All spaces surrounding the growth plate were completely filled in black color using the “Edit>Fill” tool. All images were processed in this way.

**Fig. S1:**
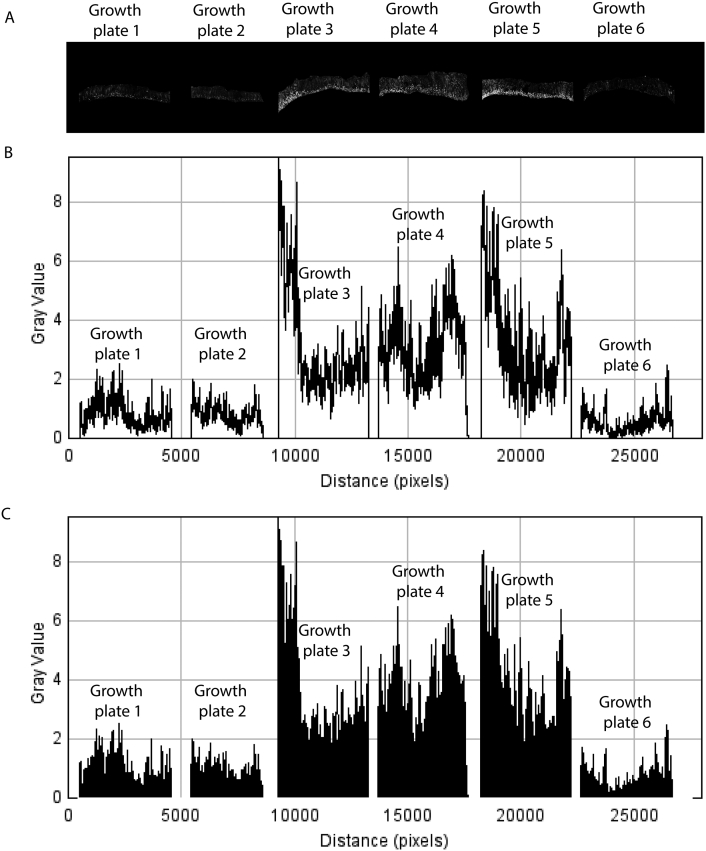
Semi-automated quantification of fluorescent images using ImageJ freeware. Details are outlined in the Materials and methods section. Brightness and contrast have been adjusted in image A for visual purposes.

Secondly, the images were stitched together in a single file (for direct comparison of the signal between the samples). To do this a new ImageJ file was created (File + N) and all images to-be-analyzed were copied and pasted into this new single image from left to right (and the order noted) leaving a gap between each image (Fig. S1A).

Thirdly, every pixel was selected (Control + A) and the signal was converted to a graph using the plot profile tool (Control + K), which generates a profile image (Fig. S1B). To quantify the area under the curve for each plot, the Wand tool was used to select a plot and its area measured using the “Analyze>Measure” function. Notice, for Wand tool to select individual plots everything under the curve needs first to be manually colored black using the Wand + the fill function (Control + F) and the x-axis deleted (Fig. S1C). Finally the data were copied from the Results window and pasted to an Excel file, where each value representing fluorescent signal was normalized by the area of the corresponding growth plate.

### 4.5 *In situ* hybridization

*In situ* hybridization was performed as previously reported for *ColX* (Newton et al., 2015), and using the same protocol for *Ihh*, but with probes kindly gifted us by Dr. Tatsuya Kobayashi (Massachusetts General Hospital).

Genotyping, skeletal preparation, incorporation and visualization of BrdU, and staining for X-gal (Chagin et al., 2014), metatarsal culture (Newton et al., 2015), and collection of measurements for bone lengths and histomorphometry were performed as described (Vuppalapati et al., 2015). The immunostaining protocol used to detect phosphorylated S6 (Newton et al., 2015) was also used to detect Ki67 (using the primary antibody: Invitrogen, MA5-14520) and p4EBP1 (Cell Signaling, 236B4).

The following is the supplementary data related to this article.

## Transparency document

Transparency document. Image 1
